# Detection of Subarachnoid Hemorrhage Using CNN with Dynamic Factor and Wandering Strategy-Based Feature Selection

**DOI:** 10.3390/diagnostics14212417

**Published:** 2024-10-30

**Authors:** Jewel Sengupta, Robertas Alzbutas, Tomas Iešmantas, Vytautas Petkus, Alina Barkauskienė, Vytenis Ratkūnas, Saulius Lukoševičius, Aidanas Preikšaitis, Indre Lapinskienė, Mindaugas Šerpytis, Edgaras Misiulis, Gediminas Skarbalius, Robertas Navakas, Algis Džiugys

**Affiliations:** 1 Department of Mathematics and Natural Sciences, Kaunas University of Technology, K. Donelaičio st. 73, 44249 Kaunas, Lithuania; robertas.alzbutas@ktu.lt (R.A.); tomas.iesmantas@ktu.lt (T.I.); 2 Laboratory of Heat-Equipment Research and Testing, Lithuanian Energy Institute, Breslaujos st. 3, 44403 Kaunas, Lithuania; edgaras.misiulis@lei.lt (E.M.); gediminas.skarbalius@gmail.com (G.S.); robertas.navakas@lei.lt (R.N.); algis.dziugys@gmail.com (A.D.); 3 Health Telematics Science Institute, Kaunas University of Technology, K. Donelaičio st. 73, 44249 Kaunas, Lithuania; vytautas.petkus@ktu.lt; 4 Center for Radiology and Nuclear Medicine, Vilnius University Hospital Santaros Klinikos, Santariskiu st. 2, 08661 Vilnius, Lithuania; alina.barkauskiene@gmail.com; 5 Department of Radiology, Lithuanian University of Health Sciences, Eiveniu st. 2, 50009 Kaunas, Lithuania; vytenisra@gmail.com (V.R.); saulius.lukosevicius@lsmu.lt (S.L.); 6 Clinic of Neurology and Neurosurgery, Faculty of Medicine, Vilnius University, M. K. Ciurlionio st. 21, 03101 Vilnius, Lithuania; danas911@gmail.com; 7 Clinic of Anaesthesiology and Intensive Care, Faculty of Medicine, Vilnius University, M. K. Ciurlionio st. 21, 03101 Vilnius, Lithuania; lapinskiene.indre@gmail.com (I.L.); mindaugas.serpytis@santa.lt (M.Š.)

**Keywords:** feature selection, parametric rectified linear unit, region-growing method, sand cat swarm optimization algorithm, stacked convolutional neural network, subarachnoid hemorrhage detection, wandering strategy, water waves dynamic factor

## Abstract

**Objectives**: Subarachnoid Hemorrhage (SAH) is a serious neurological emergency case with a higher mortality rate. An automatic SAH detection is needed to expedite and improve identification, aiding timely and efficient treatment pathways. The existence of noisy and dissimilar anatomical structures in NCCT images, limited availability of labeled SAH data, and ineffective training causes the issues of irrelevant features, overfitting, and vanishing gradient issues that make SAH detection a challenging task. **Methods**: In this work, the water waves dynamic factor and wandering strategy-based Sand Cat Swarm Optimization, namely DWSCSO, are proposed to ensure optimum feature selection while a Parametric Rectified Linear Unit with a Stacked Convolutional Neural Network, referred to as PRSCNN, is developed for classifying grades of SAH. The DWSCSO and PRSCNN surpass current practices in SAH detection by improving feature selection and classification accuracy. DWSCSO is proposed to ensure optimum feature selection, avoiding local optima issues with higher exploration capacity and avoiding the issue of overfitting in classification. Firstly, in this work, a modified region-growing method was employed on the patient Non-Contrast Computed Tomography (NCCT) images to segment the regions affected by SAH. From the segmented regions, the wide range of patterns and irregularities, fine-grained textures and details, and complex and abstract features were extracted from pre-trained models like GoogleNet, Visual Geometry Group (VGG)-16, and ResNet50. Next, the PRSCNN was developed for classifying grades of SAH which helped to avoid the vanishing gradient issue. **Results**: The DWSCSO-PRSCNN obtained a maximum accuracy of 99.48%, which is significant compared with other models. The DWSCSO-PRSCNN provides an improved accuracy of 99.62% in CT dataset compared with the DL-ICH and GoogLeNet + (GLCM and LBP), ResNet-50 + (GLCM and LBP), and AlexNet + (GLCM and LBP), which confirms that DWSCSO-PRSCNN effectively reduces false positives and false negatives. **Conclusions**: the complexity of DWSCSO-PRSCNN was acceptable in this research, for while simpler approaches appeared preferable, they failed to address problems like overfitting and vanishing gradients. Accordingly, the DWSCSO for optimized feature selection and PRSCNN for robust classification were essential for handling these challenges and enhancing the detection in different clinical settings.

## 1. Introduction

The sudden breaking of an aneurysm resulting in bleeding inside the subarachnoid space around the spinal cord and brain is called Subarachnoid Hemorrhage (SAH), where this space is generally filled with the colorless and clear fluid named cerebrospinal fluid [1,2]. Based on the cause, the SAH can be categorized into two types, traumatic and spontaneous. Spontaneous SAH causes 20% of acute cerebrovascular disorders due to hypertension, intracranial aneurysms, and spinal/brain arteriovenous malformations [3,4,5]. Spontaneous SAH is usually caused by the rupture of an intracranial aneurysm and by a diversity of other conditions like meningitis, malignant tumors, moyamoya disease, issues related to anticoagulation therapy, encephalitis, homological abnormalities, and brain vasculitis [6,7]. Traumatic SAH is the existence of blood in the subarachnoid space caused by a traumatic head/brain injury and is usually accompanied by a brain contusion. Arterial dissection, increase in intravascular pressure, damage in small arteries or veins, and direct blood extravasation are the major causes of traumatic SAH [8,9,10].

The computed tomography imaging modality is effective in the detection of SAH compared to other medical techniques like ultrasound, magnetic resonance imaging, positron emission tomography, etc. The NCCT images are effective in detecting bleeding in brain regions. In relation to other imaging techniques, the NCCT images are highly interactive and effectively find the bleeding, even when the leaked blood amount is small [11,12]. In SAH detection, the NCCT images include benefits like higher sensitivity to blood and less scanning time. The clinician’s diagnosis of SAH is based on the properties of lesions in the NCCT images [13,14,15]. However, it is difficult to obtain an accurate estimation of bleeding volume in the patients, particularly in the NCCT images. To overcome this problem, several automated models are designed related to machine learning and deep learning (DL) models [16,17]. The existing research faces many challenges such as failure to extract an appropriate feature, existence of irrelevant features, and ineffective training during the detection of the SAH grade. The addressing of aforementioned issues is considered a motivation of this research. Therefore, three pre-trained models such as GoogleNet, VGG-16, and ResNet50 were used for the effective acquisition of a wide range of patterns and irregularities, fine-grained textures and details, and complex and abstract features. Next, an optimum feature subset from an extracted feature was discovered using DWSCSO which aided in reducing the time and classifier’s complexity. The classification stability of PRSCNN was enhanced by avoiding the vanishing gradient issue based on the incorporation of PReLU.

In order to estimate the amount of leaked blood, i.e., the hemorrhage region, in a more acceptable time interval, a novel automated DL approach with feature selection was designed in the present article.

The major contributions are pointed out as follows:

- Proposed a modified region-growing for segmenting the regions affected by SAH. Modified region-growing is a simple and efficient segmentation method. The seed points were not positioned precisely in the conventional region-growing method when the background had similar color and texture to the object of interest. It was overcome in the modified region-growing by selecting the seed points based on the orientation and intensity threshold values.
- Integrated three pre-trained models (GoogleNet, VGG-16, and ResNet50) for extracting features from the segmented images. In that, GoogleNet, VGG-16, and ResNet50 were used to extract the wide range of patterns and irregularities, fine-grained textures and details, and complex and abstract features to enhance the SAH grade classification.
- The use of high-level (semantic content and visual patterns) and low-level (color, texture, contrast, and edges) features decreased the semantic space between the vectors, which increased the success rate of SAH grade classification.
- DWSCSO was developed for reducing the dimensions of extracted features; this enabled a significant reduction in the training time and complexity of the classifier. The water waves dynamic factor (WWDF) and wandering strategy were included in the DWSCSO for an effective adaptation to the complex operations and for increasing the exploration capacity.
- The Parametric Rectified Linear Unit (PReLU) Stacked Convolution Neural Network model was used for stable SAH grade classification by avoiding the vanishing gradient issue. This PReLU was chosen because it offers smoother gradient flow by backpropagation that used to achieve stable training by avoiding the vanishing gradient issue.

The present article is organized in this manner: the papers related to the topic “SAH detection” are surveyed in Section 2. The detail about DWSCSO-PRSCNN-based grade classification of SAH, outcomes, and the conclusion are specified in Section 3, Section 4, and Section 5, correspondingly.

## 2. Literature Survey

The existing articles related to SAH detection are briefly reviewed in this section. Mansour, R.F and Aljehane, N.O [14] developed the DL-based intracranial hemorrhage (ICH) approach for optimal segmentation with the Inception Network. The segmentation over the CT images was accomplished by using Kapur’s thresholding with Elephant Herd Optimization (EHO). The features from the segmented portions were obtained using DL-based Inception v4 network while multilayer perceptron was used to perform the classification. The identification of optimum threshold using EHO enhanced the segmentation. The redundant features were included in the overall feature set subjected to affect the classification performances. Wang et al. [18] used XGBoost model with the ‘xgboost’ package from R version 3.6.1 to predict the outcomes of aneurysmal SAH patients. The early prediction performed by the XGBoost assists physicians in strengthening the clinical care and therapeutic strategies for aneurysmal SAH patients. In this study, the input data were collected from 351 aneurysmal SAH patients, who were admitted to a hospital in West China. From the collected data, 70% was utilized for model training and the residual 30% was utilized for model testing. When compared with logistic regression, the XGBoost obtained better results, but this model was quite sensitive to the outliers. Furthermore, Mohammed et al. [19] presented three systems for ICH detection with different methods and materials. In the first system, the low-dimensional vectors were extracted from the CT images by employing two global descriptors. The extracted low-dimensional vectors from the Gray Level Co-occurrence Matrix (GLCM) and Local Binary Pattern (LBP) were integrated with the high-level vectors from AlexNet, ResNet-50, and GoogleNet. Further, the feature dimensions were minimized by implementing the Principal Component Analysis (PCA) technique. In the second system, AlexNet, ResNet-50, and GoogleNet models were initially employed to extract vectors from the CT images, which were further given to the Support Vector Machine (SVM) to classify the feature maps. The third system was developed by employing only AlexNet, ResNet-50, and GoogleNet. Different evaluation measures revealed that the presented systems attained more promising results in ICH detection than the existing systems. However, the use of only pre-trained models increased the computational cost of the system.

In the context of SAH detection, Rau et al. [20] used a decision tree classifier for predicting isolated traumatic SAH patients with high mortality risk, but the traditional machine learning classifier (decision tree) includes two major issues, i.e., outliers and overfitting. Malik, P [21] presented the stacked deep model classifier which has numerous layers of neural networks where every layer was developed to obtain appropriate levels of features. These features were used to obtain an improved representation learning during the classification. However, an existence of inadequate features was subjected to affect the classification. Additionally, Lee et al. [22] developed an efficient deep learning classifier for detecting acute ICH and classifying the subtypes of ICH (no hemorrhage, epidural, subdural, intra-ventricular, and intra-parenchymal hemorrhages) in the CT images. The developed system not only showed comparable performance in ICH subtype classification but also in ICH detection (while aggregating various hemorrhages). Imran et al. [23] used U-Net for detecting the subtypes of ICH. In that literature, the U-Net model’s efficacy was validated utilizing evaluation measures like recall, F1-score, and precision. The results confirmed that the U-Net model achieved superior results in ICH subtypes classification compared with the comparative models (logistic regression, stacked CNN, and SVM). However, a huge number of parameters in the U-Net model leads to an overfitting problem.

A new architecture (combination of CNN with a Recurrent Neural Network (RNN)) was created by Liu et al. [24] for detecting and classifying the subtypes of ICH in the CT images. A new loss function was utilized in the developed architecture to neglect the label dependencies that help in resolving the problem of imbalanced data distribution. The experiments performed on a benchmark dataset showed the efficacy of the developed architecture over the traditional models. However, training the CNN-RNN model is a complex task and it leads to vanishing gradient problems while using a standard activation function. Kärkkäinen et al. [25] presented an unsupervised framework based on mixture models for classifying the healthy and hemorrhaged tissues. The performance of the presented framework was validated on a public dataset, which has different hemorrhage types with various intensities and sizes. In this study, the presented framework’s results were compared with different supervised and unsupervised models. The outcomes confirmed that the developed framework significantly improved more than the existing methods by means of dice score. Furthermore, Sage and Badura [26] implemented a double branch ResNet-50 model for detecting the subtypes of ICH. Compared with the random forest and SVM classifiers, the double-branch ResNet-50 model achieved higher classification results in light of detection accuracy and F1-score, but the double-branch ResNet-50 model had high complexity. Correspondingly, Barros et al. [27] presented a stacked CNN model for effective SAH segmentation in the CT images. Initially, trivial background voxels and trivial voxels were excluded from the collected CT images, and then new hemorrhage patches were generated with a sagittal plane by performing flipping. The generated hemorrhage patches were passed to the stacked CNN model to exclude the background patches with lower classification errors. One of the main drawbacks of the CNN model in SAH segmentation was the need for an enormous number of labeled images to effectively train the model, which was a time-consuming process and computationally expensive.

An automated model was implemented by Li et al. [28] for the effective segmentation and detection of SAH. Firstly, the posterior intersection points, brain boundaries, and anterior intersection points were computed from the CT images. Secondly, vectors were extracted concerning the computed points, and further, the feature vectors were passed to the Bayesian decision model to classify the grades of SAH. Generally, the Bayesian decision model generates an enormous number of parameters during the computationally expensive classification. In addition to this, Shahzad et al. [29] developed an effective deep-learning framework for detecting and segmenting aneurysms in SAH patients on the CT images. The developed framework has obtained only comparable segmentation performance on the CT images with artifacts. Sun et al. [30] presented a Deformable Attention U-Shaped Network (DAUN) for precise segmentation of SAH. A region boundary-aware loss optimizer was utilized in the DAUN to improve the accuracy of segmentation on the smaller lesions and uneven edges. Additionally, a supervised learning approach was used for training the DAUN to balance the position and semantic information of every pixel. The experiments carried out on the Monuseg dataset demonstrate the effectiveness of the DAUN over traditional models, but it has a high processing time.

Nagaraju, S et al. [31] implemented a Transfer Learning Ensemble (TLE) framework which was used to detect and classify the intracranial hemorrhage. The developed TLE was developed by incorporating different classifiers, such as XceptionNet, InceptionV3, Resnet50, VGG19, Desnet121 and VGG16. Subsequently, a voting classifier was utilized for identifying the best classifier with enhanced accuracy. However, appropriate features were required to be extracted for further enhancement of the classification performances. Korra, S et al. [32] developed a fully connected separable convolutional network which helped the clinician at the preliminary stage of the treatment process by obtaining the discriminative feature vectors from various layers. This research considered various data augmentation approaches such as shifting, brightness adjustment, scaling, horizontal flipping, and rotation for generating the numerous image variations that improve the generalization capacity during the classification. The optimum feature subset was essential for avoiding the irrelevant features, because it was subjected to cause misclassification.

SS [33] presented the deep learning classifier to perform the detection and classification of intracranial hemorrhage based on the stacked generalization ensemble approach. The ensemble approach was designed based on five transfer learning classifiers such as InceptionResNetV2, EfficientNetB0, NASNetMobile, InceptionV3, and MobileNetV2. Subsequently, soft voting was used to add the evaluated probabilities from the individual classifier. The developed ensemble approach used a meta-learning algorithm to learn how to effectively combine the estimations from the deep learning classifiers. The deep learning classifiers in the ensemble approach were used to learn the feature hierarchies, i.e., both the low- and high-level features. However, this stacked generalization ensemble approach failed to consider the optimum feature subset, because the features with redundant information were subject to affect the performances.

Taking into account the considered models and their features, a novel automated deep learning method/model is proposed in this article for comparably precise and efficient SAH segmentation and classification of SAH grades.

The issues found from the existing research are mentioned as follows: inadequate feature extraction, existence of irrelevant features, and inefficient training during the classification. The aforementioned issues are addressed in this research based on the following strategies: The combination of GoogleNet, VGG-16, and ResNet50 were used to obtain different level of features including a wide range of patterns and irregularities, fine-grained textures and details, and complex and abstract features. Irrelevant features were removed by DWSCSO, which helps to reduce dimensions of extracted features to enhance classification. Further, the smoother gradient flow obtained by backpropagation of PReLU in PRSCNN helped to avoid the vanishing gradient issue during classification.

## 3. Methods

**Figure 1:**
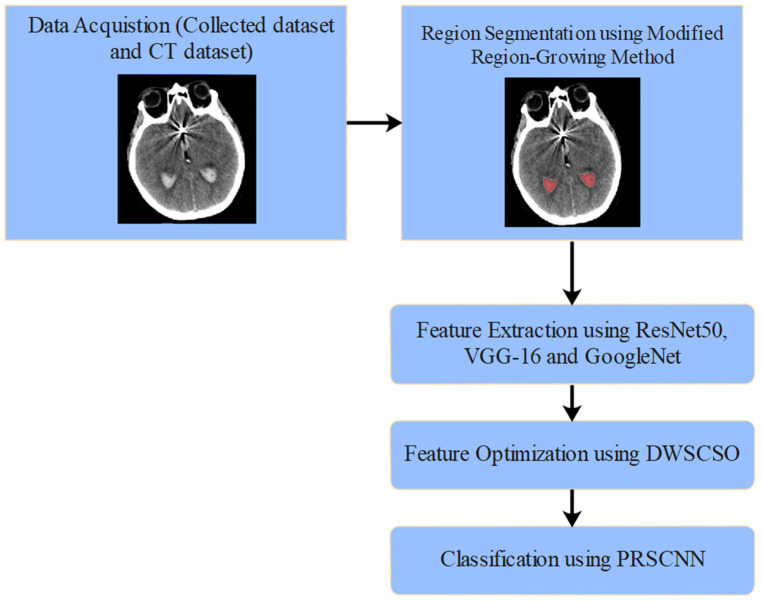
Automated SAH classification using DWSCSO and PRSCNN.

The proposed automated SAH detection model includes five phases such as NCCT image collection (from clinical data sources, where various artifacts persist), region segmentation (applying modified region-growing method), feature extraction (using ResNet50, VGG-16, and GoogleNet), feature optimization (applying DWSCSO algorithm), and SAH grade classification (using the PRSCNN model). The automated SAH classification using DWSCSO and PRSCNN is represented in Figure 1.

### 3.1. Data Description

This research was analyzed in two different datasets: collected dataset and CT dataset [34].

#### 3.1.1. Collected Dataset

**Figure 2:**
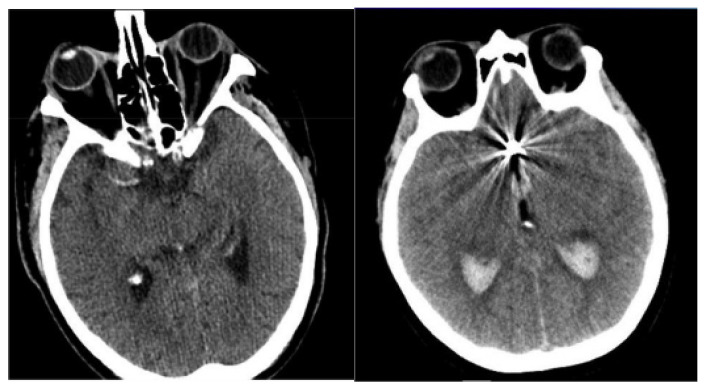
Sample-acquired NCCT images.

The NCCT brain images of 49 SAH patients were acquired from two distinct university hospitals in Lithuania. Patient inclusion criteria were: older than 18 years, diagnosed cerebral aneurysm rupture, initial NCCT scans performed routinely at patient admission. In order to increase the sample size, the collected NCCT brain images are augmented and can be used to mark regions of interest, specifically highlighting areas indicative of SAH. The image augmentation was performed by employing flipping, shifting, and random rotation techniques to generate more training images, as performing calculations with limited data samples in the deep learning models leads to overfitting problems. The use of image augmentation techniques reduces data overfitting, prevents data scarcity, and improves the model’s efficiency in image segmentation and classification. In total, 1400 NCCT images were generated, of which 1120 images were used in a training set, and the remaining 280 images were used in a testing set. The sample-acquired NCCT images are given in Figure 2.

#### 3.1.2. CT Dataset

The CT dataset for hemorrhages was acquired from the Near East Hospital in Cyprus. This dataset had 7032 CT images collected from 18 patients who had cerebral hemorrhages and 27 people who did not have cerebral hemorrhages. Accordingly, the dataset had 4343 healthy images and 2689 hemorrhagic images. The acquired images from the dataset were processed under the modified region-growing method to obtain the region of hemorrhage.

### 3.2. Region Segmentation

The region-growing method is one of the most efficient region-centric-based segmentation methods. It is also known as a pixel-based segmentation that involves seed point selection. In this case, the regions of seed pixels were grown by adding similar neighborhood pixels [35]. In conventional region-growing, the seed points are selected based on an intensity threshold value. The deviation in the intensity or noise causes over-segmentation or holes. To overcome the above-stated concern in the modified region-growing method, the seed points are selected based on the intensity and orientation threshold values [36,37]. In this segmentation, cross validation was utilized in the training set to fine-tune the threshold values. A various candidate threshold was analyzed by performing the regions of interest’s segmentation, and the segmentation performance was computed using the Dice Similarity Coefficient (DSC) and Jaccard Index (JI). The respective threshold values, which returned a higher DSC and JI, were taken as optimal thresholds. Additionally, the bootstrapping method was used to generate confidence intervals in the selected thresholds, confirming that the chosen values were statistically reliable and reproducible over diverse datasets. This helped to lessen over-segmentation and confirmed a reliable performance. The morphological operation was utilized in segmentation to avoid issues related to the unwanted regions. The steps involved in the modified region growing method are pointed out below, as follows:

- Initially, the image gradients were computed using the sobel operator. Here, the gradient states the rate of change in pixel values in NCCT images. In the context of region growing, the sobel operator utilizes two convolution kernels to identify the variations in both the vertical and horizontal directions. These two kernels compute the gradient magnitude and direction for every pixel in an NCCT image. The region growing criteria were defined according to the gradient magnitude, and the gradient direction was used to guide the direction of region expansion.
- Then, the NCCT images were segmented into different grids, $S_{i}$, based on the orientation threshold, $O_{th}$, and intensity threshold, $I_{th}$. Here, the sobel operator and histogram analysis were used to identify the $O_{th}$ and $I_{th}$, respectively. The sobel operator was used to identify the edges by evaluating the pixel’s gradient magnitude and orientation. Generally, it was used to discover the image intensity variation. This operator computed the level of intensity variation (magnitude) and direction of orientation. Equations (1) and (2) are used to compute the gradient $\left(E_{ij}\right)$ by the sobel operator.

(1) $$E_{ij}=\frac{e_{ij}}{e_{max}\;}$$

(2) $$e_{ij}=\sqrt{G_{x}^{2}+G_{y}^{2}}$$

where $e_{\max}=\max(e_{ij})$, and the gradient value of components at x and y directions are represented as $G_{x}$ and $G_{y}$, respectively.

**Figure 3:**
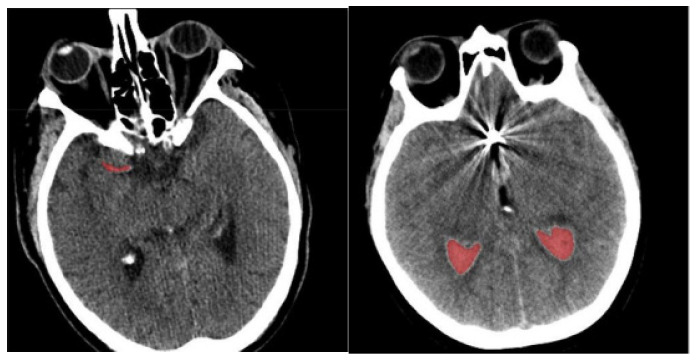
Sample-segmented images.

- The process is followed with respect to grids $S_{i}$, until the total number of grids is similar to the number of grids in the NCCT images. This is conducted as follows:
  1. In the grids $S_{i}$, compute the histogram value $L_{h}$ of every pixel;
  2. Select the frequency histogram value $F_{h}$ of the $S_{i}^{th}$ grid;
  3. Select a pixel based on the frequency histogram value $F_{h}$ and assign a respective pixel as the seed point containing the orientation value $O_{p}$ and intensity value $I_{p}$;
  4. Then, consider the adjacent pixels containing the orientation $O_{n}$ and intensity $I_{n}$;
  5. Finally, determine the differences in orientation and intensity of the pixels n and p using Equations (3) and (4). The sample-segmented images are graphically presented in Figure 3, where the red area denotes the segmented portions.

(3) $${\mathrm{df}}_{\mathrm{orientation}}=\left\|O_{n}-O_{p}\right\|$$

(4) $${\mathrm{df}}_{\mathrm{intensity}}=\left\|I_{n}-I_{p}\right\|$$

The process of segmentation using modified region-growing is important, because it mainly impacts the capacity of feature extraction and classification because the poor segmentation causes noise or irrelevant details which affect classification. A precise segmentation separated the hemorrhagic regions from the images and confirmed that only appropriate areas were transferred to feature extraction. This helped in extracting the significant features that lead to the enhancement of the capacity of classifying the SAH grade. After performing the segmentation, the isolated portions were given as input to the feature extraction where the pre-trained models were utilized to extract appropriate features.

### 3.3. Feature Extraction

In this section, the feature extraction was performed utilizing three pre-trained models: ResNet50 [38], VGG-16 [39], and GoogleNet [40] to extract high-level and low-level vectors from the segmented regions. Specifically, the inception modules and pooling operations of GoogleNet were used to obtain the wide range of patterns and irregularities that exist in the NCCT images that were helpful in identifying the delicate irregularity. The VGG-16 extracted the fine-grained textures and details using its convolutional filters and deep layers. Further, the complex and abstract features were obtained using the residual connections of ResNet50.

ResNet50: It includes 50 CNN layers (1 average-pool layer, 1 max-pool layer, and 48 convolutional layers) for feature extraction. The residual neural network is an Artificial Neural Network (ANN), which creates networks by stacking several residual blocks.

VGG-16: It has 3 dense layers, 5 max-pool layers, and 13 convolutional layers to extract vectors from the segmented NCCT images. The VGG-16 sums up to 21 layers, but only 16 weighted layers are utilized for learning the parameters.

GoogleNet: It has 22 deep layers, and it works based on the so-called inception module. The GoogleNet model uses inception modules, and it allows the network to choose between several convolutional filter sizes in every block.

By utilizing the feature-averaging technique, the feature vectors from the ResNet50, VGG-16, and GoogleNet are combined. In total, 8728 vectors were obtained from segmented NCCT images, which were processed with the SCSO algorithm for optimizing the extracted vectors.

### 3.4. Feature Optimization

The feature vectors extracted from the NCCT images were passed to the DWSCSO algorithm for optimization. In that, the WWDF of dynamic factor was incorporated to effectively adapt to the complex operations and enhance the possibility of discovering optimum solution. The wandering strategy included a triangle walk scheme and Lévy flight (LF) was used to improve the robustness of exploration capacity. Currently, the SCSO is an efficient metaheuristic-based optimization algorithm, which works on the concept of swarm intelligence and mimics the sand cat’s hunting behavior [41,42]. The wild sand cat searches or attacks the prey based on the prey’s sound frequency, because every sand cat is impressible to sound frequency. In this algorithm, the initialization matrix was generated based on the size of the extracted vectors.

#### 3.4.1. Exploration Phase (Searching for Prey)

The sand cat’s position is represented as Pos, and it even senses the prey below 2 kHz frequency. In conventional SCSO, a $r_{G}$ linearly decreases from 2 to 0, however it does not adapt well to the difficult multivariate functions. Hence, the WWDF factor is used for considering the advantage of water wave dynamics, thus it adapts to complex operations and enhances the capacity of discovering the optimum solution. The utilization of water wave’s dynamics supports the population for searching over the extensive area, minimizing the blindness of remaining individuals, improving data exchange and learning among populations, population diversity maintenance, and avoiding local optima issue. Meanwhile, the control factor k is included for handling the magnitude decrement of $r_{G}$ and it is expressed in Equation (5). The parameter R in Equation (6) controls the exploitation and exploration ability of the DWSCSO algorithm [43].

(5) $$r_{G}=2\times s\times \exp{\left(\frac{-t}{T}\right)}^{k}\times r$$

(6) $$R=2\times r_{G}\times rand\left(0,1\right)-r_{G}$$

where the random integer is denoted as s; random function is r∈[0,1]; k∈[1,3]; maximum number of iterations is denoted as T; the present iteration number is stated as t; and the value of $S_{M}=2$.

While searching a prey, every sand cat identifies a new position within its sensitivity range r, and it contributes to the exploitation and exploration algorithms. The parameter r is different for every sand cat that avoids falling into the local minima trap, and parameter r is represented in Equation (7). The parameter $r_{G}$ is used for guiding the parameter r.

(7) $$r=r_{G}\times rand\left(0,1\right)$$

The sand cat searches the prey position based on the parameter r, the current position ${Pos}_{c}(t)$, and optimal candidate position ${Pos}_{bc}$, and it is represented in Equation (8).

(8) $$Pos\left(t+1\right)=r\times \left({Pos}_{bc}\left(t\right)-rand\left(0,1\right)\times {Pos}_{c}\left(t\right)\right)$$

A triangle walk scheme is included for the sand cat to move around as it reaches its prey. The distance L1 is computed among the sand cat and its prey and computes the range of step size L2. Accordingly, it determines the walking direction of the sand cat using the Equations (9)–(13).

(9) $$L_{1}={Pos}_{bc}\left(t\right)-{Pos}_{c}\left(t\right)$$

(10) $$L_{2}=rand()-L_{1}$$

(11) β=2×π×rand()

(12) $$P=L_{1}^{2}+L_{2}^{2}-2\times L_{1}\times L_{2}\times \cos\left(\beta \right)$$

(13) $$Pos_{new}={Pos}_{bc}\left(t\right)+r\times P$$

where the location obtained via the walking scheme is denoted as $Pos_{new}$.

#### 3.4.2. Exploitation Phase (Attacking Prey)

The distance ${Pos}_{rnd}$ between the prey and sand cat is computed utilizing Equation (14) for simulating the process of attacking prey. Let us consider that the sand cat’s range of sensitivity is a circle, and the direction of motion utilizes the Roulette Wheel Selection (RWS) scheme for selecting the random angle α. The random angle is chosen among 0° and 360° and its value ranges between −1 and 1. Further, the prey is attacked based on Equation (15).

(14) $${Pos}_{rnd}=|rand\left(0,1\right)\times {Pos}_{bc}\left(t\right)-{Pos}_{c}(t)|$$

(15) $$Pos\left(t+1\right)={Pos}_{bc}\left(t\right)-r\times {Pos}_{rnd}\times cos(\alpha )$$

LF is an enhanced approach that adds randomness to the exploitation phase. LF provides a random wandering method with a step length supporting the Lévy distribution. Sometimes, the LF has higher step length, so for making it consistent with the behavior of the sand cat, it is multiplied by the constant (C=0.35). This makes the sand cat walk as near as possible. The walking strategy using LF is expressed in Equation (16).

(16) $$Pos_{new}={Pos}_{bc}\left(t\right)+\left({Pos}_{bc}\left(t\right)-{Pos}_{c}\left(t\right)\right)+r\times P$$

By regulating the parameters R and $r_{G}$, the algorithm controls the exploitation and exploration capability. If R is less than or equal to one, the prey is attacked by the sand cat, otherwise, the sand cat searches for the prey. This scenario is mathematically presented in Equation (17), and DWSCSO ends once it reaches the maximum iterations.

(17) $$Pos\left(t+1\right)=\left\{\begin{array}{cc} r\times \left({Pos}_{bc}\left(t\right)-rand\left(0,1\right)\times {Pos}_{c}\left(t\right)\right) & \left|R\right|>1;exploration\\ {Pos}_{b}\left(t\right)-{Pos}_{rnd}\times cos(\alpha )\times r & \left|R\right|\leq 1;exploitation \end{array}\right\}$$

In this algorithm, the accuracy from K-nearest neighbor (KNN) is considered as the fitness function for selecting the important features, and the time complexity is based on the sand cat’s population size and number of iterations. The DWSCSO’s time complexity is presented in Equation (18).

(18) $$O\left(\mathrm{DWSCSO}\right)=O\left(\mathrm{defined}\;\mathrm{parameters}\right)+O\left(\mathrm{location}\;\mathrm{update}\right)+O(\mathrm{population}\;\mathrm{initialization})$$

**Figure 4:**
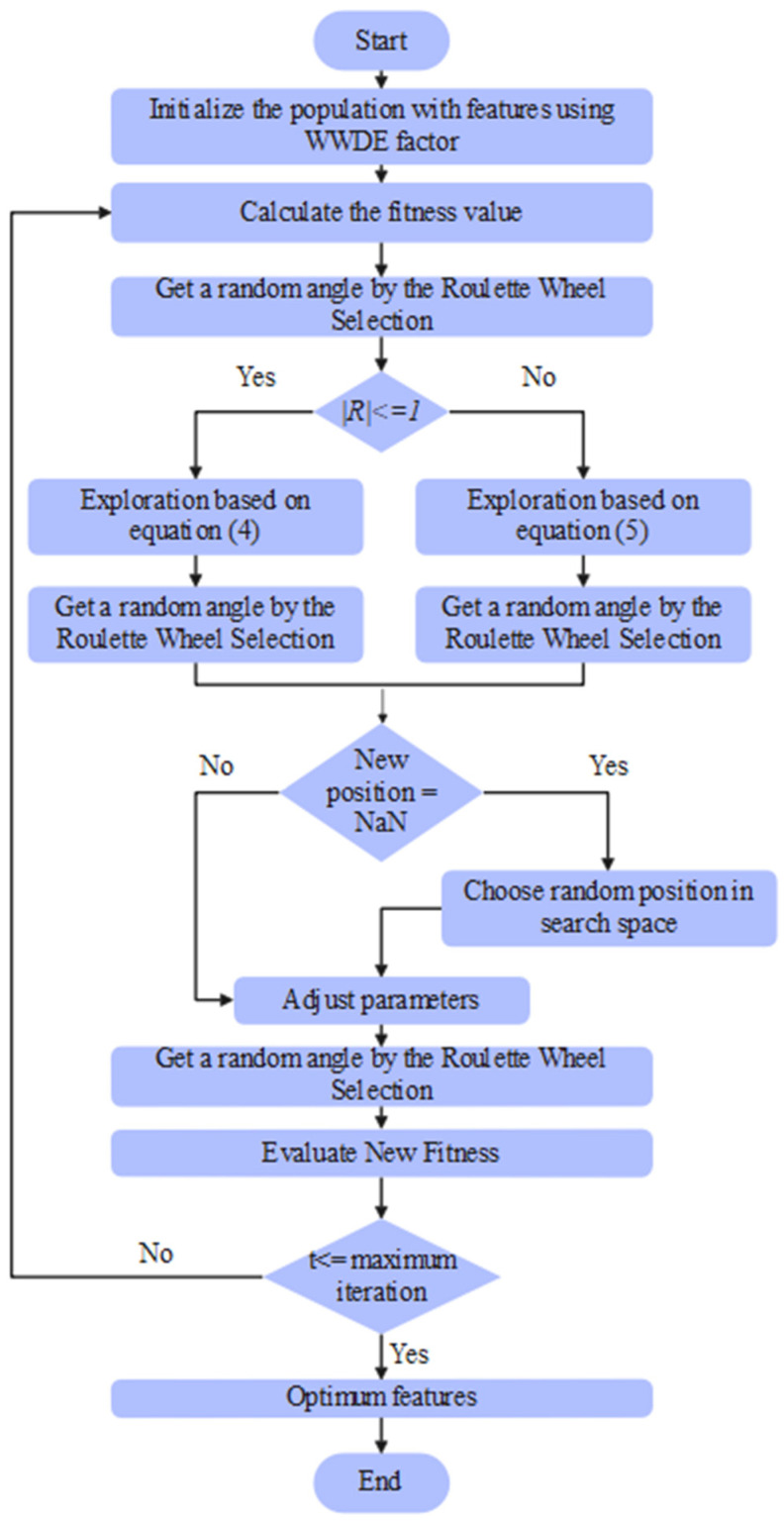
Flowchart of the DWSCSO-based feature optimization.

The parameters considered in the DWSCSO are listed as follows: size of population was 100, RWS was [0, 360], $S_{M}$ was 2, and total iterations were 100. From the extracted 8728 vectors, the DWSCSO algorithm selected 5290 vectors, which were passed to the PRSCNN model for SAH grade classification. The pseudocode and flowchart of the DWSCSO-based feature optimization is depicted in Algorithm 1 and Figure 4.
**Algorithm 1** Pseudocode of the DWSCSO algorithm
//Step 1: Initialization
Input: Maximum iterations *T*, population size *N*, fitness function *F*, WWDF, α, *r*, *r_G_*
//Initialize population *P* of *N* search agents (feature vectors)
For each search agent *i* in *P* do search solution space
  Solution space states the range of probable feature values for every agent.
End
//Step 2: Estimate the Initial Fitness
For every search agent *i* in *P* do
  Calculate fitness *F_i_* for agent *i* using the fitness function *F*
  *F_i_* = *Accuracy*(*knn_classifier*(*X_train*, *y_train*))
End
//Step 3: Main Loop—Iterate over Maximum Iterations *T*
For iteration *t* = 1 to *T* do
  //Step 3.1: For every search agent, accomplish exploration or exploitation
  For every search agent *i* in *P* do
    //Step 3.1.1: Select a random angle α for direction of movement (0° ≤ α ≤ 360°)
  α = *random_angle*() //Randomly selected using RWS.
    //Step 3.1.2: Identify if exploration or exploitation is to be executed
     If |*r*_*G*_| > 1 then //Exploration phase
      //Step 3.1.2.1: Exploration—Move to a new position using Triangle Walk Scheme
      *new_position_X_i_* = *X_i_* + *WWDF* ∗ *r* ∗ *cos*(α) ∗ *random_step*()
     Else //Exploitation phase
      //Step 3.1.2.2: Exploitation—Move using LF
      *new_position_X_i_* = *X_i_* +*levy_flight*() ∗ (*best_position* − *X_i_*)
      //LF generates a random step based on Lévy distribution.
 
     End
    //Step 3.1.3: Verify if new position is valid
     If new_position_*X_i_* is invalid (e.g., NaN, Inf, out-of-bounds) then
      //Reset the agent’s position to a valid random location in the search space
       *new_position_X_i_* = *random_valid_position*()
     End
    //Step 3.1.4: Evaluate the fitness of the new position
    *new_fitness_X_i_* = *evaluate_fitness*(*new_position_X_i_*)
    //Fitness function estimates the new feature vector for classification accuracy.
    //Step 3.1.5: Update agent’s position and fitness if the new position is better
     If *new_fitness_F_i_* > *current_fitness_F_i_* then
       *X_i_* = *new_position_X_i_*//Update the agent’s position
       *F_i_* = *new_fitness_F_i_* //Update the agent’s fitness
     Else
       Retain current position *X_i_* and fitness *F_i_*
     End
  End
  //Step 3.2: Convergence Check—Monitor improvement
  If no substantial improvement in fitness after *X* consecutive iterations then
    //Enhance the step size in LF to escape local optima and encourage exploration
     *WWDF* = *WWDF* ∗ 1.5 //enhance an exploration factor to cover more search space
  End
  //Step 3.3: Adjust parameters dynamically
  Adjust *r* and *r_G_* based on the iteration number *t*
End
//Step 4: Return the best feature vector
Determine an agent with greatest fitness score
Return best_position (best feature vector) and corresponding fitness

#### 3.4.3. Error Handling and Edge Case Considerations

The handling of error and edge case considerations are used for confirming the robustness and common applicability of the optimization algorithm. DWSCSO is developed with the following strategies for addressing the convergence issues, local minima traps, and invalid inputs.

##### Convergence Issues

The WWDF dynamically adjusts in the iterative process to eliminate inactivity during the optimization, which was used to confirm that the population remains to explore different areas of the solution space. The WWDF factor improves the adaptability of DWSCSO in difficult and higher dimensional search spaces. Moreover, the LF is used for incorporating randomness to the exploitation to avoid premature convergence. Therefore, dynamic alteration avoids the inactivity and confirms the convergence towards the global optimum.

##### Local Minima Traps

The DWSCSO developed with the triangle walk scheme and LF was used to avoid the local minima risk. Premature convergence was avoided by avoiding the repetitive patterns using the triangle walk scheme. Moreover, the risk of local optima is additionally reduced by continuously altering the angle of approach and attack using RWS.

##### Invalid Inputs Handling

The DWSCSO verifies the input ranges for confirming that the given inputs are in the boundaries or not. If the values are not set based on requirements, the default values are allocated in the optimization. The fitness function computes the feature vector quality chosen in the optimization process. Moreover, if a population faces an invalid position, the agent position is returned to a randomly chosen valid position in the search space. The fitness estimation function is also developed for handling an edge case by confirming all values are constrained within satisfactory boundaries. The fitness function evaluates the updated position. Next, the fitness function eliminates the errors that interrupt the optimization to confirm that the inputs transferred to the fitness function are valid. If any location returns to NaN or Inf, the population is initialized again utilizing a random position in exploration.

##### Outlier Detection and Robustness

The fitness function integrates outlier detection by evaluating the fitness values over the population. The DWSCSO considers the probability of either invalid input or local minima trap when the outlier is identified, i.e., fitness of population is lesser or higher than the average of population. In these situations, the exploration is enabled, making the affected population set its location based on random searches in a wide area of solution space. This confirms that the DWSCSO is robust even in the presence of corrupted or noisy data.

Therefore, regarding the incorporation of error-handling mechanisms and edge case considerations, the DWSCSO is developed to make it robust and adaptable in an extensive range of optimization tasks. Subsequently, the selected features from the DWSCSO are given as an input to the PRSCNN to accomplish SAH grade classification.

### 3.5. SAH Grade Classification

The selected 5290 vectors were given to the PRSCNN for SAH grade classification. The PRSCNN model was designed with distinct layers such as an input layer, convolutional layer, PReLU activation layer, pooling layer, and flattened/fully connected layer [44,45,46]. The incorporated PReLU had smoother gradient flow due to the backpropagation process. This smoother flow of gradient leads to stable and effective training which helps to avoid the vanishing gradient issue. The PReLU has the ability to allow the negative values in the training process, which helps to offer a better depiction of the data. Accordingly, it was used to enhance the generalization of unseen data. A brief description of the layers are discussed below, as follows:

Input layer: The selected 5290 vectors are fed to the input layer, and it is represented as a three-dimensional matrix. The dimension is W×H×D, where W is represented as width, H is denoted as height, and D is indicated as depth, where the depth corresponds to the color channels.

Convolutional layer: The convolutional layer computes the output of the nodes, which are interconnected to the local regions of the matrix. Here, the dot product is computed between the values related to an input: local region and a set of weights (filter).

PReLU activation layer: The PReLU function represented in Equation (19) is used to avoid the issue of dying ReLU and it performs well with the negative inputs as well as allows backpropagation. The incorporation of negative parts as input offers reliable predictions. Moreover, it helps to avoid the vanishing gradient issue.

(19) $$f\left(x_{i}\right)=\left\{\begin{array}{c} x_{i},\;\;\;\;\;\;\;\;\;\;\;\;if\;x_{i}>0\\ \alpha_{i}x_{i},\;\;\;\;\;\;\;\mathrm{otherwise} \end{array}\right.$$

where $x_{i}$ denotes only one element from vector x; input vector is denoted as x; and learnable parameter is denoted as $\alpha_{i}$.

Pooling layer: Uses down-sampling technique to reduce the height and width of the convolved features.

Fully connected or flattened layer: The convolved features are fed to the flattened layers here. The class probabilities are calculated and outputted in a three-dimensional array with dimensions of 1×1×K, where *K* is the number of classes (four grades). Grade I is depressed consciousness level or focal deficit, grade II is mild alteration, grade III is severe headache, and grade IV is mild headache.

The parameters of the PRSCNN model are the initial learning rate, which is 0.001, the loss function is log loss, the drop factor of learning rate is 0.2, the drop period of learning rate is 5, the maximum epochs is 100, the minimum batch size is 500, the activation function is ReLU, the optimizer is Adam, and the momentum rate is 0.9.

## 4. Results and Discussion

The proposed modified region-growing method and DWSCSO-PRSCNN model are simulated using Python 3 on a system with the specifications of a windows operating system, Intel core i10 12th-generation processor, Santa Clara, CA, USA, and 64 GB random access memory. The modified region-growing method’s effectiveness was analyzed using evaluation measures like the Jaccard Index (JI), Dice Similarity Coefficient (DSC), PA, and MPA. Correspondingly, the classification model’s (DWSCSO-PRSCNN) effectiveness was analyzed using evaluation measures like accuracy, Matthews Correlation Coefficient (MCC), and F1 score on a collected dataset where an 80:20 ratio is considered for training and testing purposes.

### 4.1. Evaluation Measures

The explanation about the undertaken evaluation measures (JI, DSC, PA, MPA, accuracy, MCC, and f1 score) is detailed in this subsection. The JI is determined as the area of intersection between the ground truth region and segmentation region and divided by the area of union between the ground truth region and segmentation region. Additionally, the DSC efficiently calculates the overlap among the ground truth and segmentation region by performing intersection over union between two sets, where A indicates a segmented region by performing the modified region-growing method and B represents ground truth region. The mathematical formulas of JI and DSC are depicted in Equations (20) and (21).

(20) $$\mathrm{JI}=\frac{\mathrm{TP}}{\mathrm{TP}+\mathrm{FP}+\mathrm{FN}}=\frac{\left|A\cap B\right|}{\left|A\cup B\right|}$$

(21) $$\mathrm{DSC}=\frac{2\mathrm{TP}}{2\mathrm{TP}+\mathrm{FP}+\mathrm{FN}}=\frac{2|A\cap B|}{\left|A\right|+|B|}$$

Similarly, the PA is determined as the ratio of precisely segmented pixels divided by the total pixels. For instance, the PA of K+1 classes (background and K foreground classes) is mathematically denoted in Equation (22). PA can be treated as a semantic segmentation metric, which represents the percentage of pixels which are precisely classified in an image. In addition, the MPA is determined as the ratio of precisely segmented pixels divided by the average of total pixels, and it is mathematically specified in Equation (23).

(22) $$\mathrm{PA}=\frac{\sum_{i=0}^{K}p_{ii}}{\sum_{i=0}^{K}\sum_{j=0}^{K}p_{ij}}$$

(23) $$\mathrm{MPA}=\frac{1}{K+1}\sum_{i=0}^{K}\frac{p_{ii}}{\sum_{j=0}^{K}p_{ij}}$$

The evaluation measure: Accuracy estimates how many times a classification model (DWSCSO-PRSCNN) made a correct prediction in the entire collected dataset. F1 measures a classification model’s (DWSCSO-PRSCNN) accuracy by combining the recall and precision. Additionally, the Matthews correlation coefficient (MCC) considers all four values (True Negative (TN), True Positive (TP), False Positive (FP), and False Negative (FN)) in the confusion matrix to estimate the efficacy of the classification model. The MCC ranges between −1 and 1, where −1 represents completely wrong multiclass classification and 1 indicates correct multiclass classification. The expressions to compute accuracy, F1 score, and MCC are represented in Equations (24)–(26).

(24) $$\mathrm{Accuracy}=\frac{\mathrm{TP}+\mathrm{TN}}{\mathrm{TP}+\mathrm{TN}+\mathrm{FP}+\mathrm{FN}}\times 100$$

(25) $$\mathrm{MCC}=\frac{\mathrm{TP}\times \mathrm{TN}-\mathrm{FP}\times \mathrm{FN}}{\sqrt{\left(\mathrm{TP}+\mathrm{FP})(\mathrm{TP}+\mathrm{FN})(\mathrm{TN}+\mathrm{FP})(\mathrm{TN}+\mathrm{FN}\right)}}\times 100$$

(26) $$F1\;\mathrm{Score}=\frac{2\mathrm{TP}}{\mathrm{FP}+2\mathrm{TP}+\mathrm{FN}}\times 100$$

### 4.2. Quantitative Analysis Related to Segmentation

The simulation results of different segmentation methods for collected and CT datasets are depicted in Table 1 and Table 2. The FCM clustering, Otsu thresholding, K-means clustering, superpixel clustering and region-growing are considered for the evaluation of modified region-growing, because all these approaches are parameter sensitive. Viewing the tables shows that the modified region-growing method achieved precise segmentation results on a collected dataset with a JI of 0.94, DSC of 0.95, PA of 0.93, and MPA of 0.90, which are better than comparative segmentation approaches like Fuzzy C Means (FCM) clustering, Otsu thresholding, K-means clustering, and the region-growing method. The clustering based approaches mainly depend on the predefined clusters and initialized centroids. Accordingly, the clustering based segmentation returns the less defined edges, due to the complexity in the identification of fine details and edges. On the other hand, the modified region-growing approach is effective for the images with indefinite textures and intensities, therefore it adaptively enlarges the region according to the pixel similarity instead of depending on the predefined clusters. Consequently, the developed modified region-growing leads to precise delineation of structures even with the complex images. Therefore, an integration of contextual information from adjacent pixels helps to obtain more effective segmentation than the clustering approaches.

If the original NCCT images have clear edges, the modified region-growing method provides good segmentation results. Additionally, the modified region-growing method precisely separates the regions, which have similar properties, compared with the traditional segmentation methods. The modified region-growing method consumed a minimal processing time of 6.32 s for region segmentation in the collected dataset. The comparative segmentation methods—FCM clustering, Otsu thresholding, K-means clustering, and the region-growing method—consumed processing times of 11.32 s, 10.11 s, 15.44 s and 8.20 s in the collected dataset, respectively.

### 4.3. Quantitative Analysis Related to Feature Extraction

This section provides an analysis about the combination of all models, i.e., GoogleNet, VGG-16, and ResNet50 used in the feature extraction with the individual model’s comparison to know the effectiveness of the combined model. Additionally, some state-of-the-art approaches such as VGG-19 and SqueezeNet developed using Python 3.7, Keras 2.3 and TensorFlow 1.5 were used for analysis. The combined model was the CNN model, so the aforementioned CNN models were used in the comparison. Table 3 and Table 4 provide the results analysis of feature extraction approaches for collected and CT datasets, respectively. Moreover, the GoogleNet, VGG-16, VGG-19, SqueezeNet, ResNet50, and a combined model (GoogleNet + VGG-16 + ResNet50) utilized the computational time of 8 ms, 15 ms, 16 ms, 13 ms, 10 ms, and 33 ms per image of collected dataset. This analysis demonstrated that the combination of all three models provides a more enhanced performance in SAH grade classification than the individual models. The computational time of the combined model was higher when it was analyzed with individual model. However, the combination of GoogleNet, VGG-16, and ResNet5 helps to obtain the wide range of patterns and irregularities, fine-grained textures and details, and complex and abstract features for enhancing the SAH grade classification. The reason for not selecting a less resource-intensive method like SqueezeNet is that it offers less computational costs, but it does not obtain the fine-grained details and complex abstractions that are required for effective SAH detection.

An important trade-off when considering the combination of GoogleNet, VGG-16, and ResNet50 is the increase in computational complexity that causes a higher memory usage, training time, and processing time in both the training and inference phases. However, the obtained performance enhancement from this combined model in clinical task justifies the trade-off in computational time. The developed DWSCSO in feature selection minimizes the overall feature dimension by around 40% and that helps to minimize the training time and complexity.

### 4.4. Quantitative Analysis Related to Classification

**Figure 5:**
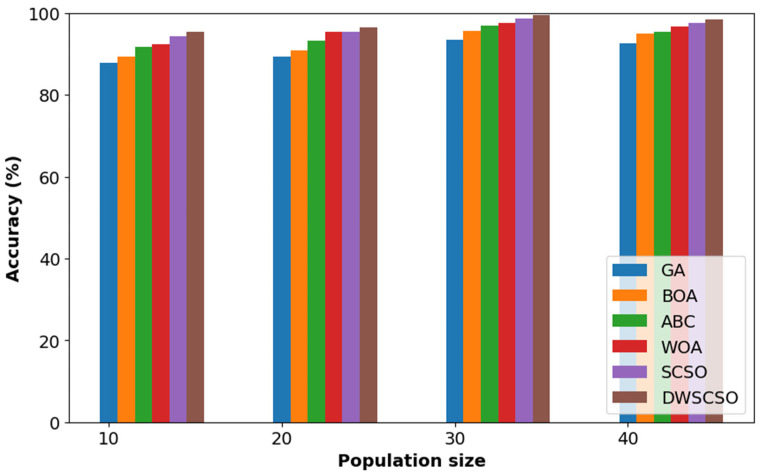
Analysis of optimization with different size of population for collected dataset.

**Figure 6:**
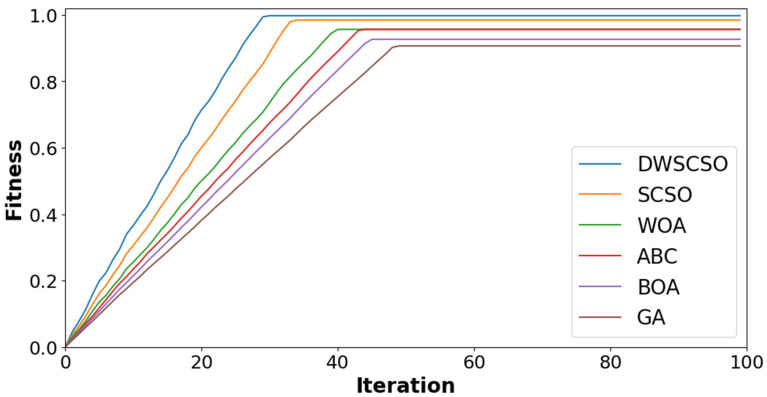
Analysis of convergence for collected dataset.

In this research, the important contribution was to perform feature selection using the DWSCSO. Therefore, the developed DWSCSO was analyzed with different optimization algorithms like the Genetic Algorithm (GA), Butterfly Optimization Algorithm (BOA), Artificial Bee Colony (ABC), Whale Optimization Algorithm (WOA), and SCSO. At first, the optimization algorithm was analyzed with different sizes of population, such as 10, 20, 30 and 40, as shown in Figure 5 for the collected dataset. Based on Figure 5, it was concluded that the optimization algorithm with a population size of 30 provided enhanced classification results. On the other hand, the convergence analysis for the collected dataset was performed as depicted in Figure 6. The developed DWSCSO had improved convergence more than the GA, BOA, ABC, WOA and SCSO. The dynamic factor of WWDF was incorporated for adapting according to the complex operations and enhanced the probability of identifying the optimum solution. On the other hand, the wandering strategy was utilized to enhance the exploration capacity. Therefore, both the WWDF and wandering strategy used in the DWSCSO helped to improve the convergence.

The simulation results of the different optimization algorithms with a PRSCNN model were depicted in Table 5 and Table 6 for the collected and CT datasets, respectively. By investigating Table 5 and Table 6, the combination of the DWSCSO algorithm with the PRSCNN model achieved maximum classification results compared with the comparative optimization algorithms. The feature dimensionality reduction or selection of discriminative vectors reduced the training time of the PRSCNN model to 32.22 s for the collected dataset. The DWSCSO achieved an accuracy of 99.48% for the collected dataset that was higher than the remaining optimization algorithms. The higher accuracy over the classification was obtained due to an effective search of the optimum features by the DWSCSO. The water wave dynamics from WWDF was used to search over an extensive area for optimum features and enhanced the population diversity maintenance while the LF was used to enhance the robustness of the exploration capacity of features.

The computational complexity of the DWSCSO for the collected dataset was analyzed in terms of computational time with GA, BOA, ABC, WOA, and SCSO, as shown in Table 7. This evaluation demonstrated that the DWSCSO had lesser computational time due to its improved convergence obtained by the incorporation of WWDF and wandering strategy in the DWSCSO. Moreover, the statistical test, i.e., the Friedman test, was used for the proposed DWSCSO with the collected dataset, as shown in Table 8. This test denoted that the DWSCSO had better rank than the optimization methods. The *p*-value represents the significant difference among the evaluated methods.

The PRSCNN is a deep learning classifier, therefore it is evaluated with some state-of-the-art deep learning classifiers such as the decision tree, Graph Convolutional Network (GCN), Artificial Neural Network (ANN), Autoencoder, and Convolutional Neural Network (CNN). The simulation results of different classification models are presented in Table 9 and Table 10 for the collected and CT datasets, respectively. Here, the analysis is performed for actual feature vectors and optimized feature vectors using the DWSCSO algorithm. The parameters considered in the comparative classification models are pointed out below, as follows:

- Decision tree (criterion is Gini, splitter is best, and maximum depth is 30);
- GCN (layer is 3, hidden size is 64 and dropout rate is 0.2);
- ANN (learning number is 13, learning rate is 0.001, and target error is 0.001);
- Autoencoder (dropout rate is 0.5, epoch is 100, batch size is 128, and learning rate is 0.001);
- CNN and PRSCNN (layer is 7, kernel size is 3 × 3, filters/channels per layer is 128, pooling size is 2 × 2, learning rate = 0.001, batch size = 32, num epochs = 10, and regularization weight = 0.0001).

The accuracy of PRSCNN over 10 multiple runs for the collected dataset was 99.45%, 99.47%, 99.48%, 99.47%, 99.49%, 99.51%, 99.46%, 99.53%, 99.49%, and 99.46% where the standard deviation was 0.0234. Moreover, the accuracy of PRSCNN with augmentation for the collected dataset was 99.48% while without incorporating the data augmentation, it was 96. 18%, which was lower due to the overfitting issue. By examining tables, the combination of the DWSCSO algorithm with the PRSCNN model obtained higher classification results in the collected dataset with a classification accuracy of 99.48%, MCC of 99.53%, and F1 score of 99.48%, respectively. The obtained classification results were higher than the existing classification models such as the decision tree, GCN, ANN, Autoencoder, and CNN. Next, the analysis of PReLU with different activation functions such as the Rectified Linear Unit (ReLU), Leaky ReLU, and Exponential Linear Unit (ELU) is given in Table 11 and Table 12 for the collected and CT datasets, respectively. This result demonstrates that the PReLU had a better performance than the ReLU, Leaky ReLU, and ELU.

In this article, the PRSCNN model efficiently captures the patterns and spatial relationships in the NCCT images. The PRSCNN model learns complex features by stacking several convolutional and pooling layers resulting in higher classification accuracy. The computational time of ReLU, Leaky ReLU, ELU, and PReLU are 0.1 ms, 0.2 ms, 0.5 ms, and 0.5 ms per image, respectively. However, the PReLU obtains smoother gradient flow by backpropagation which helps to obtain stable training for mitigating the vanishing gradient issue. The PReLU has the capacity of processing negative values which additionally helps to obtain reliable prediction. PReLU has a better performance because it learns the negative values during the training, offering enhanced flexibility and adaptability that helps to discover the subtle dissimilarities in SAH grades.

**Figure 7:**
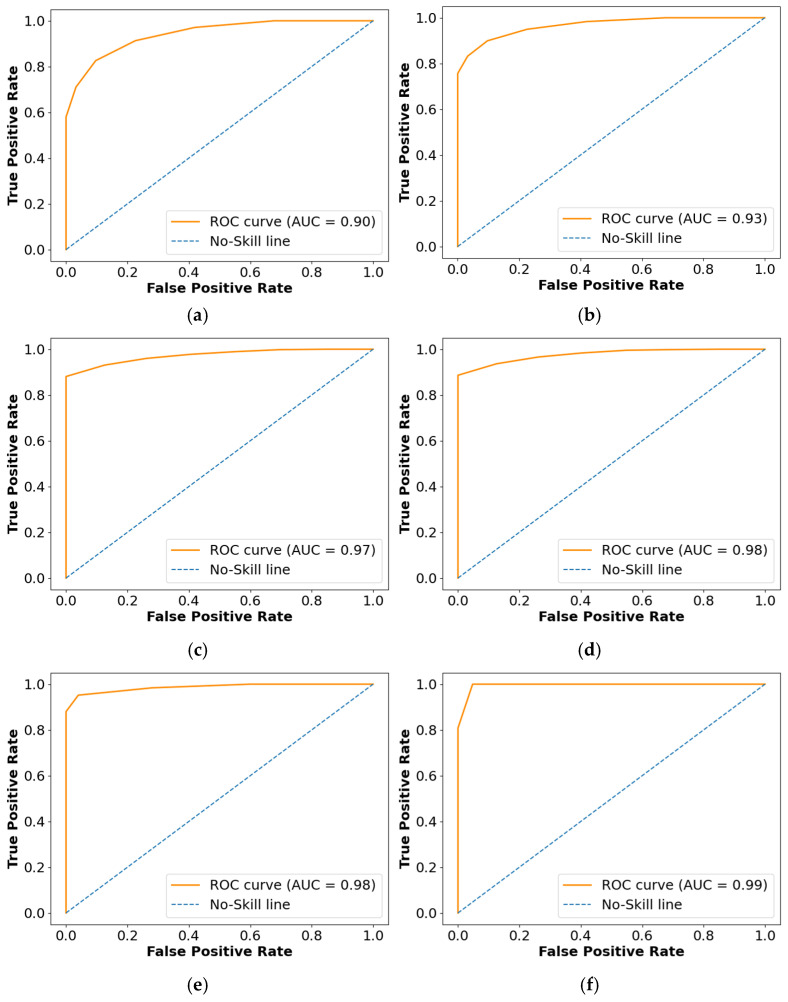
ROC curve for collected dataset, (**a**) decision tree, (**b**) GCN, (**c**) ANN, (**d**) Autoencoder, (**e**) CNN, (**f**) PRSCNN.

**Figure 8:**
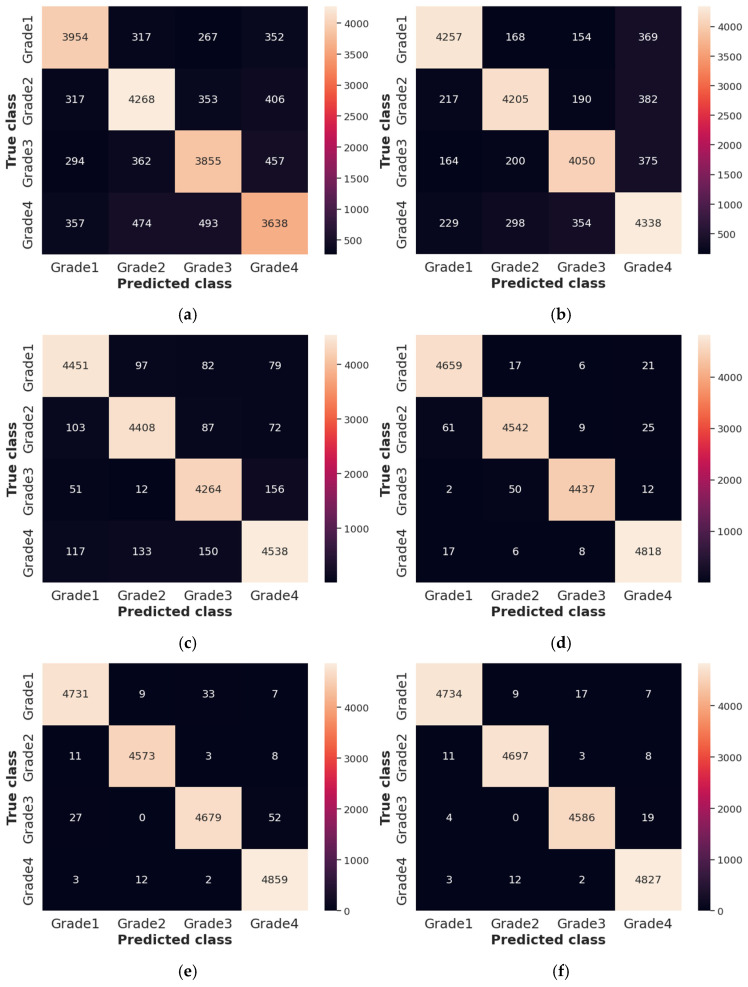
Confusion matrix for collected dataset, (**a**) decision tree, (**b**) GCN, (**c**) ANN, (**d**) Autoencoder, (**e**) CNN, (**f**) PRSCNN.

The Receiver Operating Characteristics (ROC), Area Under the Curve (AUC), and confusion matrix for the collected dataset are shown in Figure 7 and Figure 8, respectively. In ROC curve, the blue dashed line denotes the no-skill line that is used to evaluate the performance of developed classifier, while orange line denotes the ROC curve of developed classifier. The ROC curve was utilized for the classification issued in various threshold settings while the AUC denoted the degree of separability measure. On the other hand, the confusion matrix was utilized to summarize the identification of the classification issue. The ROC is a probability curve which was used to determine the level of differentiating among the classes. The ROC curve of Figure 7 represents that PRSCNN has a higher AUC, hence it provides better classification than the other classifiers. Moreover, the confusion matrix of PRSCNN depicts that it has lesser misclassification when compared with the decision tree, GCN, ANN, Autoencoder, and CNN. The misclassification in PRSCNN occurred because some samples from the collected dataset had lesser variation between the different grade images.

**Figure 9:**
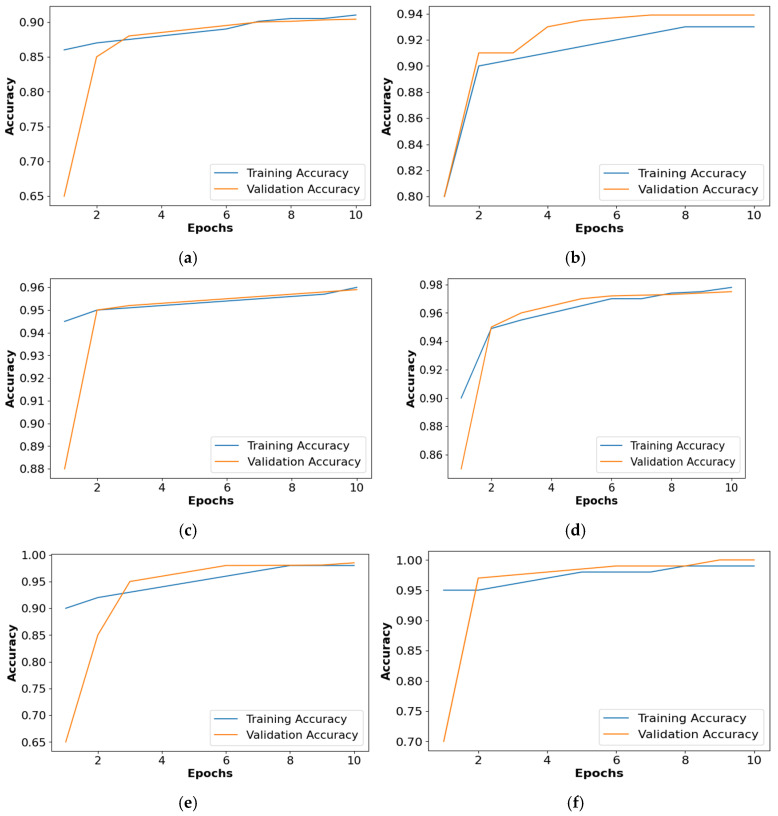
Accuracy graph for collected dataset, (**a**) decision tree, (**b**) GCN, (**c**) ANN, (**d**) Autoencoder, (**e**) CNN, (**f**) PRSCNN.

Figure 9 shows the accuracy comparison of the collected dataset for all the classifiers. Based on Figure 9, it is confirmed that the PRSCNN had better classification than the decision tree, GCN, ANN, Autoencoder, and CNN. Moreover, the issue of overfitting in PRSCNN was minimized by incorporating the DWSCSO-based feature selection.

**Figure 10:**
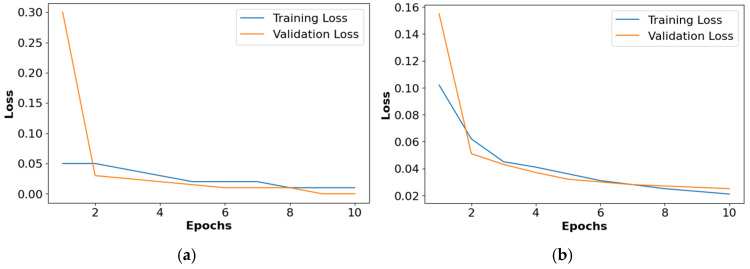

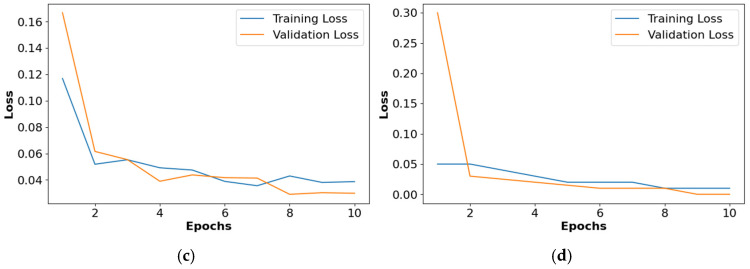
Loss graph for collected dataset, (**a**) ReLU, (**b**) Leaky ReLU, (**c**) ELU, (**d**) PReLU.

The loss graphs for the different activation functions such as ReLU, Leaky ReLU, ELU, and PReLU are shown in Figure 10. This analysis confirms that the PReLU had a better performance than the ReLU, Leaky ReLU, and ELU.

Additionally, the DWSCSO-PRSCNN model was analyzed using K-fold cross validations. In the context of image classification, cross-fold validations provided a robust estimation of the DWSCSO-PRSCNN model’s performance by assessing its generalization ability across various data subsets. This process decreased the overfitting risk to a particular training–testing split. By inspecting Table 13 and Table 14, the proposed DWSCSO-PRSCNN model achieved high classification outcomes in five-fold cross validation (80%:20% training and testing) than the other cross fold validations: two-fold (50:50% training and testing), four-fold (75:25% training and testing), and eight-fold (87.50%:12.50% training and testing).

Edge cases such as hemorrhages with uneven shapes or those positioned near anatomical boundaries cause extra difficulties in the modified region-growing segmentation approach. This segmentation has the complexity in differentiating among the delicate variations in intensity and cause over-segmentation of missed detections. In the scenario of edge cases, the model sensitivity was observed to reduce by 2.1%, mainly due to the misclassification of adjacent structures as hemorrhages. However, the overall performance still remained higher, and these situations emphasized areas for additional improvement in the segmentation process.

### 4.5. Quantitative Analysis Related to Noisy Images and Handling of Missing Values

Table 15 shows the analysis of the DWSCSO-PRSCNN with noise simulated and noiseless images for further justifying the effectiveness. Noise in the medical images affected the capacity of the model while obtaining the meaningful features, specifically in NCCT images of the brain. NCCT images are vulnerable to artifacts such as low contrast, motion blur, and scanner noise among the affected regions and healthy tissue. These issues affect the segmented region quality that leads to the affecting of the soloing feature extraction. The modified region-growing method generates the over-segmented or under-segmented regions when the input has higher levels of noise. For instance, noise causes the model to discover false positives by discovering portions that appear identical to the hemorrhagic regions. Accordingly, an increment in the FP leads to a decrease in accuracy from 99.68% to 97.32%.

The performance of the PRSCNN for the analysis of handling missing values simulated for 10% of the data is shown in Table 16. Incomplete or missing data is common in real-world clinical settings where specific images or patient information may be corrupted or unavailable. A missing value in NCCT features affects the feature selection of DWSCSO and leads to misclassification. Specifically, the sensitivity is affected, because the model failed to precisely classify hemorrhagic cases.

### 4.6. Comparative Analysis

This section provides the comparative analysis for the developed DWSCSO-PRSCNN model with a standard benchmark dataset, i.e., the CT dataset [34], where an 80:20 ratio was considered for training and testing purposes. This comparison of DWSCSO-PRSCNN with the CT dataset was performed with DL-ICH [14] and GoogLeNet + (GLCM and LBP) [19], ResNet-50 + (GLCM and LBP) [19], and AlexNet + (GLCM and LBP) [19], as shown in Table 17. The reason for choosing the aforementioned methods is that all the methods come under the category of deep learning classifiers. It demonstrates that the DWSCSO-PRSCNN outperforms better than the existing approaches. The enhanced searching capacity of DWSCSO in feature selection using the WWDF and wandering strategy helps to select an optimum feature subset for enhancing the classification. Further, the mitigation of the vanishing gradient issue and obtaining the stable training enhances the SAH grade classification.

The *p*-value for accuracy of DWSCSO-PRSCNN was less than 0.001, representing that there was a statistically significant difference among the accuracy of DWSCSO-PRSCNN and other methods. This demonstrates that variation in the performance of DWSCSO-PRSCNN and other methods is not because of random variation but denotes the actual enhancements in the DWSCSO-PRSCNN performance. The confidence interval was computed for accuracy to additionally compute the ambiguity around the mean values. For a 95% confidence level, the intervals for accuracy of DWSCSO-PRSCNN from the results, i.e., accuracy, were from 99.48% to 99.76%. This narrow confidence interval represents the higher level of confidence in the stated results, denoting the reliability and robustness of DWSCSO-PRSCNN performance. After assessing the significance via ANOVA, the Tukey’s Honest Significant Difference was applied to perform post-hoc analysis for discovering the specific groups which varied from each other. This Tukey test validated that the DWSCSO-PRSCNN significantly improved than the other methods where *p*-value was 0.001 for all pairwise evaluations. This additionally represented that DWSCSO-PRSCNN was statistically best in detecting and classifying the grade of SAH.

### 4.7. Discussion

As depicted in the earlier sections, the region segmentation of SAH and classification of SAH are integral parts of this article. A modified region-growing method was proposed in this article for precise segmentation of the regions affected by SAH. The effectiveness of the modified region-growing method is depicted in Table 1, where the proposed segmentation method not only improves the segmentation accuracy but also decreases the processing time of the segmentation. Correspondingly, in the classification phase, the combination of the DWSCSO algorithm with the PRSCNN model improved the performance of SAH grade classification more than the traditional classification models and optimization algorithms. The optimal high-level and low-level vectors (selected by the DWSCSO algorithm) were passed to the PRSCNN model for SAH grade classification. The selection of optimal high-level and low-level vectors decreased the computational time to 32.22 s and even the model complexity to linear. The efficiency of the classification model (DWSCSO-PRSCNN) was specified in Table 3, Table 4, Table 5, Table 6, Table 7, Table 8, Table 9, Table 10, Table 11, Table 12, Table 13, Table 14, Table 15, Table 16 and Table 17. The early detection of SAH assists clinicians in timely treatment and efficient therapeutic intervention, which reduces the mortality rate. Moreover, the developed DWSCSO-PRSCNN also provides better performance with the CT dataset than the DL-ICH [14] and GoogLeNet + (GLCM and LBP) [19], ResNet-50 + (GLCM and LBP) [19], and AlexNet + (GLCM and LBP) [19]. Therefore, it is confirmed that the DWSCSO-PRSCNN has better generalization in both the collected dataset and CT dataset. The developed research is clinically beneficial as it provides an automated framework for a precise estimation of blood leakage in SAH cases, which is significant for prompt and effective treatment. The development of rapid and precise segmentation and classification of SAH minimizes the dependency on manual interventions which are prone to errors and time-consuming processes. Thus, the developed research is useful in enhancing diagnostic efficiency in emergency settings, improving decision making, and possibly enhancing patient progress by confirming rapid treatment, making it appropriate for both present and upcoming clinical applications.

The DWSCSO-PRSCNN is trained and analyzed using two different datasets: the collected dataset and CT dataset. However, the changes in the imaging equipment, patient populations, and scanning protocols create differences in the dataset features, impacting the generalization of the model to unseen data. These changes are being considered in upcoming data collection. For example, the images from different hospitals show the variation between resolution, noise level, and contrast setup that leads to the increase in complexity during feature extraction. Furthermore, the issue creates a class imbalance in the dataset, specifically when handling subtle representation or rare cases of SAH. In real-world scenarios, some SAH grades are underrepresented, which makes the model become biased towards the more frequent classes.

## 5. Conclusions

This research article focuses on two problems in the SAH analysis: DWSCSO-based feature selection and the PRSCNN-based classification of SAH. A modified region-growing method was introduced for segmenting the affected regions in the collected real-time NCCT images. Optimum features were discovered using DWSCSO with the WWDF and wandering strategy. The WWDF used in DWSCSO helped to search over extensive areas for optimum features and enhanced the population diversity maintenance while the LF enhanced the robustness of exploration capacity. Next, the PReLU obtained smoother gradient flow by backpropagation, which helped to achieve the stable classification in the PRSCNN by avoiding the vanishing gradient issue. Accordingly, reliable predictions were obtained in the PRSCNN by processing the negative values. Further, the training time and overfitting risk of the classification model were reduced by selecting the optimum feature vectors. Compared with traditional segmentation methods (FCM clustering, Otsu thresholding, K-means clustering, superpixel clustering, and region growing), the modified region-growing method achieved a high JI in the collected dataset at 0.94, with a DSC of 0.95, PA of 0.93, and MPA of 0.90. The combination of the DWSCSO algorithm with the PRSCNN model achieved high classification results in the collected dataset, i.e., the F1 score was 99.48%, with a MCC of 99.53% and accuracy of 99.48%, which were better than other combinations. Additionally, the developed DWSCSO-PRSCNN also provided better performance in the CT dataset than the DL-ICH and GoogLeNet + (GLCM and LBP), ResNet-50 + (GLCM and LBP), and AlexNet + (GLCM and LBP), which proves the generalizability of detection. From the ANOVA results, post-hoc analysis, and confidence intervals, the DWSCSO-PRSCNN denoted more statistically significant enhancements than the existing methods. These outcomes offer robust proof that the DWSCSO-PRSCNN has better performance in SAH grade detection. However, the generalizability of the model is limited to the variations in imaging protocols and data from different clinical settings. Moreover, the developed PRSCNN is sensitive to noise, missing data, and class imbalance in real-world scenarios.

As a future extension, automated hyperparameter tuning, pruning inactive features, handling of missing data, and cross-domain adaptation can be developed for addressing the effectiveness of the model when processed with large-scale clinical applications. In addition, inactive feature vectors in the CNN feature extraction can be eliminated to further enhance the performance of SAH detection.

## Figures and Tables

**Table 1:** Simulation results of different segmentation methods for collected dataset.

Segmentation Methods	JI	DSC	PA	MPA
FCM clustering	0.72	0.78	0.74	0.69
Otsu thresholding	0.73	0.79	0.78	0.73
K-means clustering	0.81	0.88	0.84	0.77
Superpixel clustering	0.92	0.93	0.89	0.84
Region-growing	0.89	0.92	0.92	0.87
Modified region-growing	0.94	0.95	0.93	0.90

**Table 2:** Simulation results of different segmentation methods for CT dataset.

Segmentation Methods	JI	DSC	PA	MPA
FCM clustering	0.75	0.80	0.77	0.72
Otsu thresholding	0.79	0.82	0.83	0.77
K-means clustering	0.83	0.92	0.88	0.82
Superpixel clustering	0.94	0.95	0.92	0.88
Region-growing	0.91	0.94	0.95	0.92
Modified region-growing	0.96	0.96	0.95	0.93

**Table 3:** Simulation results of different feature extraction methods for collected dataset.

Feature Extraction Methods	Accuracy (%)	MCC (%)	F1 Score (%)	Sensitivity (%)	Specificity (%)	AUC
GoogleNet	95.27	94.12	94.67	93.64	96.12	0.952
VGG-16	96.35	95.18	95.84	94.21	97.06	0.961
VGG-19	96.81	95.67	96.33	94.76	97.53	0.963
SqueezeNet	94.00	92.85	93.40	91.71	95.19	0.940
ResNet50	97.42	96.25	96.81	96.31	98.22	0.975
GoogleNet + VGG-16 + ResNet50	99.48	99.53	99.48	98.56	99.47	0.996

**Table 4:** Simulation results of different feature extraction methods for CT dataset.

Feature Extraction Methods	Accuracy (%)	MCC (%)	F1 Score (%)	Sensitivity (%)	Specificity (%)	AUC
GoogleNet	95.67	95.69	94.91	93.50	96.85	0.957
VGG-16	96.92	96.96	96.02	94.75	97.10	0.963
VGG-19	97.30	97.35	97.15	95.00	97.80	0.965
SqueezeNet	95.42	94.18	94.50	92.70	96.30	0.946
ResNet50	98.44	97.26	97.71	96.75	98.80	0.980
GoogleNet + VGG-16 + ResNet50	99.62	99.73	99.51	98.80	99.70	0.997

**Table 5:** Simulation results of different optimization algorithms for collected dataset.

Optimization Algorithms	Accuracy (%)	MCC (%)	F1 Score (%)	Sensitivity (%)	Specificity (%)	AUC
GA	93.44	95.33	95.43	93.58	95.45	0.933
BOA	95.58	96.44	96.48	95.03	96.63	0.950
ABC	96.92	97.24	97.92	96.08	97.15	0.959
WOA	97.46	97.45	97.94	96.75	97.99	0.977
SCSO	98.68	98.10	98.63	97.82	98.15	0.981
DWSCSO	99.48	99.53	99.48	98.56	99.47	0.996

**Table 6:** Simulation results of different optimization algorithms for CT dataset.

Optimization Algorithms	Accuracy (%)	MCC (%)	F1 Score (%)	Sensitivity (%)	Specificity (%)	AUC
GA	95.40	96.01	95.35	95.12	95.07	0.939
BOA	96.09	96.79	9684	95.28	95.10	0.944
ABC	96.73	95.98	96.31	96.05	96.11	0.949
WOA	97.03	97.80	96.88	97.03	96.87	0.981
SCSO	98.76	98.27	98.85	98.12	98.35	0.986
DWSCSO	99.62	99.73	99.51	98.80	99.70	0.997

**Table 7:** Simulation results of computational time for collected dataset.

Optimization Algorithms	Computational Time (S)
GA	110.30
BOA	101.08
ABC	81.66
WOA	83.90
SCSO	78.02
DWSCSO	70.12

**Table 8:** Friedman test for collected dataset.

Optimization Algorithms	Friedman Rank
GA	2.43
BOA	3.98
ABC	4.37
WOA	5.78
SCSO	7.04
DWSCSO	7.63
*p*-value	$2.0149\times 10^{-21}$

**Table 9:** Simulation results of different classification models for collected dataset.

**Actual Feature Vectors**						
Classifiers	Accuracy (%)	MCC (%)	F1 Score (%)	Sensitivity (%)	Specificity (%)	AUC
Decision tree	88.24	87.94	88.98	87.67	88.45	0.873
GCN	89.26	90.90	90.40	89.32	90.20	0.907
ANN	92.36	93.50	92.34	92.89	93.10	0.915
Autoencoder	94.27	93.88	93.54	93.48	92.12	0.939
CNN	94.81	94.01	93.99	94.77	94.89	0.944
PRSCNN	95.22	95.90	94.90	94.09	95.40	0.955
**Optimized Feature Vectors**						
Classifiers	Accuracy (%)	MCC (%)	F1 Score (%)	Sensitivity (%)	Specificity (%)	AUC
Decision tree	90.30	93.28	92.28	93.45	93.81	0.902
GCN	93.90	95.02	93.82	94.91	94.80	0.930
ANN	95.38	97.38	98.33	96.84	96.99	0.966
Autoencoder	97.30	98.76	98.70	97.60	97.73	0.979
CNN	98.09	99.00	99.01	98.22	98.84	0.982
PRSCNN	99.48	99.53	99.48	98.56	99.47	0.996

**Table 10:** Simulation results of different classification models for CT dataset.

**Actual Feature Vectors**						
Classifiers	Accuracy (%)	MCC (%)	F1 Score (%)	Sensitivity (%)	Specificity (%)	AUC
Decision tree	89.22	89.15	89.04	89.32	89.14	0.881
GCN	90.98	90.70	91.60	90.64	91.22	0.918
ANN	93.68	93.86	92.12	93.75	93.15	0.935
Autoencoder	94.66	94.53	95.06	95.14	94.57	0.947
CNN	95.77	94.47	95.15	95.83	95.50	0.954
PRSCNN	96.54	96.16	95.72	96.43	96.82	0.962
**Optimized Feature Vectors**						
Classifiers	Accuracy (%)	MCC (%)	F1 Score (%)	Sensitivity (%)	Specificity (%)	AUC
Decision tree	91.27	91.62	91.87	91.26	91.20	0.929
GCN	92.81	93.22	92.85	93.40	92.91	0.940
ANN	94.78	95.02	95.47	95.71	94.82	0.956
Autoencoder	97.72	97.17	97.91	96.93	97.17	0.978
CNN	98.42	98.65	98.34	97.70	97.74	0.983
PRSCNN	99.62	99.73	99.51	98.80	99.70	0.997

**Table 11:** Simulation results of different activation functions for collected dataset.

Activation Functions	Accuracy (%)	MCC (%)	F1 Score (%)	Sensitivity (%)	Specificity (%)	AUC
ReLU	92.74	91.47	92.88	92.60	92.03	0.937
Leaky ReLU	95.84	94.25	94.29	94.72	94.23	0.941
ELU	96.94	97.73	96.22	97.16	96.83	0.970
PReLU	99.48	99.53	99.48	98.56	99.47	0.996

**Table 12:** Simulation results of different activation functions for CT dataset.

Activation Functions	Accuracy (%)	MCC (%)	F1 Score (%)	Sensitivity (%)	Specificity (%)	AUC
ReLU	93.45	93.54	93.56	93.51	93.91	0.942
Leaky ReLU	96.95	95.90	96.04	96.48	96.90	0.964
ELU	97.36	98.83	96.73	98.20	98.02	0.987
PReLU	99.62	99.73	99.51	98.80	99.70	0.997

**Table 13:** Different K-fold cross validation results of DWSCSO-PRSCNN for collected dataset.

Measures	K = 2	K = 4	K = 5	K = 8
MCC (%)	94.94	95.82	99.53	97.21
F1-score (%)	93.63	94.04	99.48	93.69
Accuracy (%)	97.24	98.19	99.48	96.17
Sensitivity (%)	96.34	97.33	98.56	94.67
Specificity (%)	95.11	97.67	99.47	94.62
AUC	0.959	0.961	0.996	0.950

**Table 14:** Different K-fold cross validation results of DWSCSO-PRSCNN for CT dataset.

Measures	K = 2	K = 4	K = 5	K = 8
MCC (%)	95.78	97.62	99.73	95.81
F1-score (%)	96.10	98.39	99.51	95.29
Accuracy (%)	96.66	98.58	99.62	96.28
Sensitivity (%)	95.74	97.05	98.80	94.25
Specificity (%)	95.53	96.33	99.70	94.72
AUC	0.965	0.971	0.997	0.945

**Table 15:** Simulation results of classifiers for noisy images.

Classifiers	Accuracy (%)	
	Noiseless Images	Noisy Images
Decision tree	91.27	86.53
GCN	92.81	86.74
ANN	94.78	88.21
Autoencoder	97.72	92.56
CNN	98.42	93.91
PRSCNN	99.62	97.32

**Table 16:** Simulation results of classifiers for missing data.

Classifiers	Sensitivity (%)	
	Without Missing Data	With 10% Missing Data
Decision tree	91.26	84.56
GCN	93.40	87.35
ANN	95.71	91.42
Autoencoder	96.93	92.58
CNN	97.70	93.51
PRSCNN	98.80	95.40

**Table 17:** Comparison of DWSCSO-PRSCNN.

Activation Functions	Accuracy (%)
DL-ICH [14]	95.06
GoogLeNet + (GLCM and LBP) [19]	98.9
ResNet-50 + (GLCM and LBP) [19]	99.1
AlexNet + (GLCM and LBP) [19]	99.3
DWSCSO-PRSCNN	99.62

## Data Availability

The datasets used and analyzed during the current study are available from the corresponding author upon reasonable request.
